# Distinctive Geometrical Traits of Proximal Femur Fractures—Original Article and Review of Literature

**DOI:** 10.3390/medicina59122131

**Published:** 2023-12-07

**Authors:** Christos Vlachos, Margarita Michaela Ampadiotaki, Eftychios Papagrigorakis, Athanasios Galanis, Dimitrios Zachariou, Michail Vavourakis, George Rodis, Elias Vasiliadis, Vasileios A. Kontogeorgakos, Spiros Pneumaticos, John Vlamis

**Affiliations:** 1 3rd Orthopedic Department, National and Kapodistrian University of Athens, KAT General Hospital, 14561 Athens, Greece; efpapagr@hotmail.com (E.P.); athanasiosgalanis@yahoo.com (A.G.); dimitriszaxariou@yahoo.com (D.Z.); michail.vavourakis@hotmail.com (M.V.); ilivasil@med.uoa.gr (E.V.); spirosgp@med.uoa.gr (S.P.); jvlamis@med.uoa.gr (J.V.); 2 2nd Orthopedic Department, KAT General Hospital, 14561 Athens, Greece; marab.ortho@gmail.com; 3 Department of Radiology, KAT General Hospital, 14561 Athens, Greece; rodisgeorge@gmail.com; 4 1st Orthopedic Department, National and Kapodistrian University of Athens, Attikon General University Hospital, 12462 Chaidari, Greece; vkonto@2med.uoa.gr

**Keywords:** proximal femur, proximal femoral geometry, femoral neck fractures, femoral trochanteric fractures

## Abstract

*Background and Objectives:* The incidence of proximal femoral fractures is escalating rapidly, generating a significant challenge for healthcare systems globally and, carrying serious social and economic implications. The primarily object of this study was to discover potential distinguishing factors between fractures occurring in the femoral neck and trochanteric region. *Materials and Methods:* We performed a prospective cohort study of the radiographic images of 70 people over 65 years of age who were admitted to the orthopedic department with hip fracture and who fulfilled our eligibility criteria. Neck Length (NL), Offset Lenth (OL), Hip Axis Length (HAL), Neck Shaft Angle (NSA), Wiberg Angle (WA), Acetabular Angle (AA), Femoral Neck Diameter (FND), Femoral Head Diameter (FHD), Femoral Shaft Diameter (FSD), Femoral Canal Diameter (FCD) and Tonnis classification were recorded. For the comparison of the categorical variables, Pearson’s χ2 criterion was used, while Student’s *t*-test was applied for the comparison of means of quantitative variables across fracture types. *Results:* There were no statistically significant variances observed while comparing the selected geometric parameters of the proximal femur with the type of fracture. This finding was reaffirmed in relation to age, gender, and Tonnis classification. However, a moderate correlation was noted, revealing comparatively reduced values of HAL, FHD, and FND in women as opposed to men. *Conclusions:* The inability of our research to establish the differentiative geometric factors between femoral neck and trochanteric fractures underscores the need for further investigations, which would take into consideration the intrinsic characteristics of the proximal femur.

## 1. Introduction

Hip fractures constitute a significant complication of osteoporosis, accounting for 24% of all fractures in the elderly [1,2]. These fractures are associated with high rates of mortality and morbidity, posing a substantial challenge for patients, their families and national healthcare systems [3,4,5]. To be more precise, in the United States, a patient experiencing a proximal femoral fracture incurs approximately USD 40,000 in direct treatment costs and nearly USD 5000 for subsequent rehabilitation in the forthcoming years [6,7]. It is also worth mentioning that the average age of patients experiencing their initial proximal femoral fracture is 78.5 years, and that the mean duration until the occurrence of a second fracture is 37.2 months [8]. Furthermore, the first-year mortality rate increases by 21.2% in patients after their first hip fracture [9]; however, after their second hip fracture, this percentage is escalated to 55% [10]. To amplify the scope of this problem, the growing aging population, attributed to advancements in medicine and pharmacology, has already transformed hip fractures into a prominent global health issue.

Proximal femoral fractures are primarily caused by lateral falls onto the ground [11] and are classified as either femoral neck or femoral trochanteric fractures, each requiring distinct treatment and rehabilitation approaches. Various studies have explored the predictive factors of each fracture type, yet a definitive conclusion remains elusive. An increasing body of research has affirmed that the structural configuration of hip geometry significantly influences proximal femoral bone strength [12]. Consequently, there is substantial importance in investigating the geometric parameters of the hips in elderly patients with hip fractures. Notably, our study is conducted within a European population. However, it is pertinent to acknowledge that several authors have pointed out the limited applicability of results from European and American studies to Asian or Chinese populations, citing differences in shape, size, and geometry in the proximal femur among racial groups [13,14]. Furthermore, it is remarkable that, in nearly 68% of cases involving bilateral hip fractures, the second fracture is of the same type as the initial one. Specifically, it has been observed that 62% of the fractures occurring in the femoral neck and 72% of those in the trochanteric region are preceded by a previous contralateral fracture of the same type. This discovery implies that there may exist distinct characteristics associated with femoral neck and trochanteric fractures [15,16,17].

The primary object of this study is twofold: firstly, to explore the correlation between easily quantifiable geometrical factors of the proximal femur and the type of fracture, and secondly, to develop a screening tool facilitating early preventive measures. In conducting our research, we aligned ourselves with the reasoning of various authors who have asserted that the manner and intensity of a fall on the ground do not significantly impact the type of fracture [18,19,20]. In addition, in consonance with this research focus, we conducted a comprehensive literature review to enable a comparative analysis of our findings with those reported by different authors.

## 2. Methods

### 2.1. Inclusion/Exclusion Criteria

In our prospective cohort study, we enrolled participants who met the following criteria: (i) patients aged over 65 years old and (ii) those with either a femoral neck (FNF) or a femoral trochanteric fracture (FTF) as (iii) a result of a low-energy side fall.

Conversely, we excluded individuals who had a history of the following: (i) pathological hip fractures, (ii) bilateral hip fractures, (iii) congenital femur and pelvis deformities, (iv) bone tumors or Paget disease, and (v) previous surgical interventions performed either on the same hip or the contralateral hip. Taking into consideration almost the identical inclusion and exclusion criteria, we conducted a narrative review of the extant literature pertinent to our subject. Nevertheless, we had to exclude studies in which only the abstract was available, studies conducted in languages other than English, studies utilizing quantitative CT, and studies carried out on cadaveric specimens.

The total number of participants included in the study (N) amounted to 70, comprising 51 females and 19 males. They were categorized into two groups: the FNF group (*n* = 30) and the FTF group (*n* = 40), based on X-ray findings. Each participant provided formal consent to participate in the study, with the understanding that their data would remain confidential, and they would be identified by a unique serial number.

### 2.2. Measurement Parameters

We selected 11 geometric parameters for our investigation, namely Neck Length (NL), Offset Lenth (OL), Hip Axis Length (HAL), Neck Shaft Angle (NSA), Wiberg Angle (WA), Acetabular Angle (AA), Femoral Neck Diameter (FND), Femoral Head Diameter (FHD), Femoral Shaft Diameter (FSD), Femoral Canal Diameter (FCD) and Tonnis classification. These measurements were obtained from X-rays of the healthy hip, adhering to established guidelines from the literature [21]. In more specific terms, all participants enrolled in the research underwent an anteroposterior X-ray of the pelvis and hips, wherein (i) the patient assumed a supine position; (ii) the lower extremities were rotated medially by 15–25°; (iii) the machine’s lamp was positioned one meter above the patient’s level and was centered at the midpoint between the anterior superior iliac spine and the pubis symphysis; (iv) the lateral alignment boundaries were delineated by the skin borders, while proximal limits extended to the borders of the iliac crests, and distal limits encompassed the middle third of the femur; (v) the dimensions of the detector were 35 cm × 43 cm; and (vi) the radiation exposure parameters were set at 70–80 kVp and 20–30 mAs. Moreover, to ensure accuracy and reliability, each parameter was measured by two individuals, with two measurements taken by one person and one measurement by another. It is noteworthy that the X-rays were conducted during the patient’s admission to the Emergency Department, rather than in subsequent days or during follow-up. This approach served to safeguard our measurements from osteopenia and sarcopenia, conditions commonly observed postoperatively and following prolonged immobilization [22,23].

### 2.3. Statistical Analysis

Categorical variables are described as absolute (N) and relative (%) frequencies. Quantitative variables were tested for normality of distribution using the Kolmogorov–Smirnov statistical test. The test indicated that the variables followed a normal distribution. Consequently, they are described using means ± standard deviations. For the comparison of the categorical variables, Pearson’s *χ*^2^ criterion was used, while Student’s t-test was applied for the comparison of means of quantitative variables across fracture types. Pearson’s correlation coefficient was used for bivariate correlations between age and continuous variables. All tests were two-sided. Bonferroni adjustment was used in order to control Type I error inflation due to multiple comparisons, setting the significance level to 0.003. Cohen’s kappa and Intraclass Correlation Coefficient were used for testing the interobserver and intraobserver agreement reliability. Their values were 0.84 and 0.95 (95% C.I.: 0.93, 0.97), indicating an almost perfect agreement. The statistical package Stata v.17.0 (StataCorp LLC., College Station, TX, USA) was used for the analysis.

## 3. Results

Table 1 provides an overview of the sample characteristics. In Table 2, a comprehensive analysis comparing all variables, encompassing geometric measurements, is presented, focusing on the different types of femoral fractures. As per our analytical approach, there were no notable differences observed in the measurements between the two fracture types. Additionally, when exploring the data based on fracture type, no significant variations were identified in the mean age, gender distribution, and Tönnis classification distribution.

The analysis of the data based on gender is comprehensively presented in Table 1 and Table 2. A notable observation emerges from the mean values of the hip axis length (HAL), revealing a significant contrast between women and men. Specifically, women exhibited consistently lower HAL values, reflecting a distinct anatomical characteristic. This pattern extends to the mean dimensions of the head and neck, where women’s measurements were significantly diminished compared to those of men (5.2 ± 0.3 vs. 5.7 ± 0.2, 3.7 ± 0.3 vs. 4.1 ± 0.4, *p*: < 0.001, <0.001, respectively).

Conversely, when checking the mean shaft and canal diameters, no statistically significant differences according to gender were detected (3.0 ± 0.3 vs. 3.1 ± 0.2, 1.6 ± 0.3 vs. 1.6 ± 0.3, *p*: < 0.192, <0.440, respectively), suggesting a level of gender-neutral consistency in these specific anatomical features.

Enlarging our research to consider the relationship between these measurements and age, bivariate correlations highlight that only HAL demonstrated a noteworthy and inversely proportional association with age. This relationship, though moderate in strength (−0.392, −0.425, −0.407, −0.396, *p*: 0.001, <0.001, <0.001, 0.001, respectively), underlines the intriguing dynamic that, as our patient cohort aged, the specific anatomical measurements tended to exhibit an obvious decrease. These variable findings contribute valuable insights regarding the interplay between gender, age, and anatomical characteristics in our study population.

## 4. Discussion

Hip fractures represent a substantial healthcare challenge, constituting a considerable 30% of all fragility fractures [24]. On a global scale, projections suggest that 1 in 3 women and 1 in 5 men aged 50 and above will suffer from an osteoporotic fracture during the remainder of their lifetimes [25,26]. Notably, around 51% of proximal femoral fractures are documented in Europe and America, with the remaining cases predominantly dispersed across the Western Pacific region and Southeast Asia [27]. A distinctive characteristic of this health concern is evident in the statistics from the year 2006, wherein osteoporosis was responsible for an alarming annual incidence of over 8.9 million fractures globally, equating to an osteoporotic fracture occurring approximately every 3 s. Moreover, in 2010, an estimated 158 million individuals were identified as being at a heightened risk of fractures. Projections for the year 2040 anticipate a doubling of this figure, attributable to demographic shifts [24].

Numerous authors who have attempted to delineate the predictive factors associated with proximal femoral fractures have traditionally treated them as a singular entity, neglecting to discern between the distinct categories of femoral neck and trochanteric fractures [28,29,30]. It is crucial to underscore the importance of such a differentiation, given the inherent and substantial disparities that exist in relation to risk factors, etiological considerations, morphological characteristics, as well as the spectrum of surgical interventions and rehabilitation protocols [5,31]. The recognition of these distinctive parameters holds considerable potential not only for the enhancement of surgical techniques, but also for the formulation of comprehensive strategies aimed at the prevention of subsequent fractures in the future.

**Figure 1:**
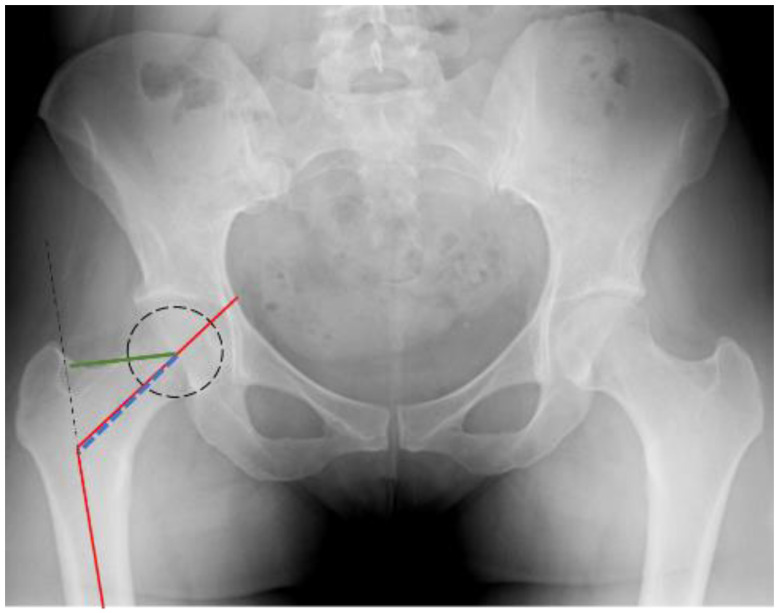
NSA, NL, OFFSET.

The Neck Shaft Angle (NSA) is considered, for multiple reasons, to be an autonomous geometric parameter of the proximal femur [32]. The main biomechanical rationale is that a wider NSA results in increased force absorption by the proximal femur in the event of a lateral fall [33]. This is defined as the point of intersection between the anatomical axis of the femoral head and neck and the anatomical axis of the femur [21] (Figure 1). The hypothesis suggests that a great NSA is associated with reduced cortical strength in the inferior portion of the femoral neck relative to the superior segment. Consequently, the application of increased bending stress could potentially result in the occurrence of a FNF [34]. It is noteworthy that the NSA undergoes constant changes throughout the aging process, regardless of skeletal maturation, ultimately leading to a decline [35]. This observation could be correlated with the fact that FTFs chiefly occur in older individuals. Moreover, the NSA notably affects the range of motion in the hip, transferring body weight to the broader base of the femoral neck. Hence, its anatomical restoration is important for a favorable surgical outcome. The fact that a larger NSA is linked to FNF is strongly supported by numerous authors [21,31,36,37,38,39]. In our study population in Greece, the average NSA for the FNF group was 133°, whereas for the FTF group, it was 130.9°, indicating no statistically significant difference. The same conclusions were also reached by Panula et al. [40] and Lima et al. [41].

**Figure 2:**
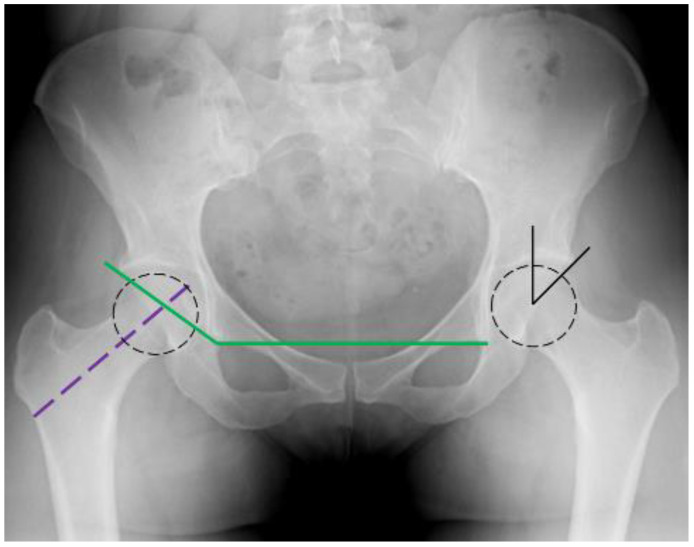
HAL, AI.

The Wiberg angle is delineated by the intersection of a line that transects the center of the femoral head and extends to the most lateral edge of the acetabulum, encompassing any osteophytes, with a line that is vertical to the center of the femoral head [29] (Figure 2). Consequently, the angle tends to increase in cases of degenerative arthritis. Previous studies conducted by Yamauch et al. [42], Cukurlu et al. [43] and Hu et al. [21] have supported the hypothesis that in the event of a side fall, the acetabular rim impinges distally on the femoral neck, exerting a substantial force on a more lateral region of the proximal femur, which can potentially lead to FTF. In addition, these studies have confirmed a noteworthy correlation between the severity of hip osteoarthritis, in terms of narrowed joint spaces and elongated osteophytes and the incidence FTF. In spite of our consideration of the Wiberg angle and the Tonnis classification of osteoarthritis, we, along with Rotem et al. [44], did not establish any association with the concrete fracture type.

The Acetabular Angle is determined in our study by the horizontal plane of the pelvis, which unions the inferior margins of the bilateral teardrops, and a second line that extends through the outermost point of the acetabular roof [45] (Figure 2). Consequently, the presence of joint osteoarthritis and osteophyte formation increases its values. In accordance with the previous mentioned assumption, in the event of a lateral fall, it is observed that the lateral area of the proximal femur comes into contact with the periphery of the acetabulum, which in this particular scenario, exhibits a more lateralized position. The force applied to sustain the hip under such circumstances could plausibly result in a FTF [43]. However, within our study sample, we did not manage to establish this association, as no statistically significant difference was observed between the groups.

The Hip Axis Length (HAL) is characterized as an independent risk factor associated with proximal femur fractures, following a similar biomechanical sceptic as the NSA. It is quantified as the linear distance extending from the lateral edge of the base of the great trochanter to the innermost margin of the pelvic rim. This straight line remains parallel to the femoral neck axis [41,46] (Figure 2). The main hypothesis in this context is that a longer HAL signifies an extended lever arm, formed between the center of the hip and the femur [36,37,47,48]. This, in turn, increases the risk of a potential hip fracture [30,49]. Specifically, Rotem et al. and patron et al. [44,50] have provided support for the theory that a longer HAL is linked to FNF. However, our study was unable to confirm this observation. Studies performed by Hu [21] et al. and Lima et al. [41] also align with this direction. On the other hand, Im et al. [51], in their study of the Korean population, noticed that people suffering from FTF tend to have a longer HAL in comparison to those with FNF.

Neck Length (NL) is defined as the distance between the anatomical axis of the femur and the central point of the femoral head. On the other hand, Offset is described as the length of a line extending horizontally from the center of the femoral head and intersecting the anatomical axis of the femur [43] (Figure 1). Both of these parameters must be properly restored during the intraoperative procedure, as this addresses the tension of the surrounding soft tissues of the hip and ensures the right length of the limb. Continuing in the same logical framework as with HAL, it can be deducted that greater values of these variables would result in an increased length of the level arm between the hip center and femur. This, in turn, would amplify the risk of a potential proximal femoral fracture. Although our study, as well as the one performed by Kazemi et al. [11], did not manage to establish a direct correlation between the values of NL and Offset and the type of fracture, Patron et al. [50] and Ferris et al. [52] demonstrated in their retrospective research that these parameters could be linked to the incidence of FNF. Once more, Im et al. [51] reported the converse findings.

**Figure 3:**
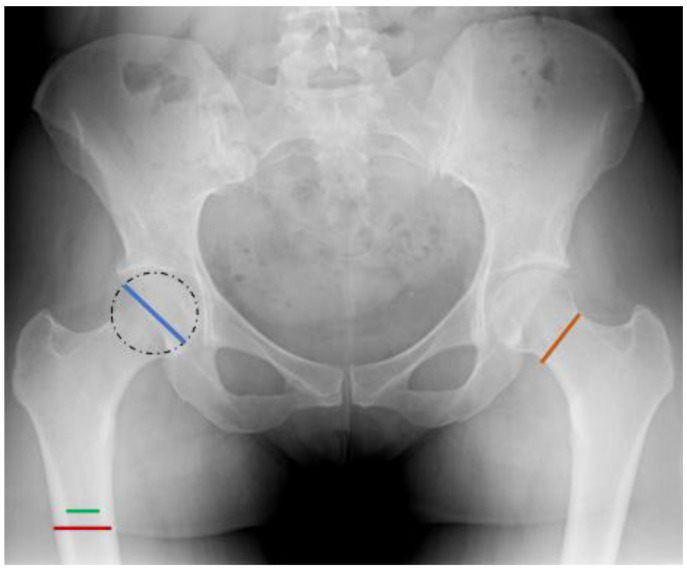
FHD, FND, FSD, FCD.

The Femoral Neck Diameter (FND) is described as the shortest distance, aligned perpendicular to the axis of the femoral neck, extending between the lateral superior margin and the medial inferior margin of the femoral neck [21] (Figure 3). The femoral neck area possesses two significant characteristics. Firstly, it lacks the presence of periosteum. Secondly, it features the calcar region, which is a thick vertical wall placed at the lower and posterior part of the femoral neck. Particularly, the calcar region remains structurally stable throughout the aging process and is unaltered by osteoporotic changes. Consequently, only at the upper half of the femoral neck does the cortical bone progressively weaken, while the trabecular bone becomes loosened, leading to the constitution of porous cavities [21,53,54,55]. This remark is supported by authors that examined the BMD in the femoral neck region [56,57]. The parameter of the FND has been a subject of doubt among authors, as certain studies suggest that shorter values are associated with FTF [21,44], while others with FNF [11,43]. Lastly, our study did not prove any important association between the FND values and type of hip fracture. This discrepancy of results indicates the necessity of further studies that will analytically investigate the internal microarchitecture of the femoral neck.

The Femoral Head Diameter (FHD) is defined as the maximum distance from the lateral superior edge to the inner inferior edge of the femoral head, assuming that it approximates a circular shape [21] (Figure 3). Although our measurements proved that FHF is higher in males, in alignment with the observations made by other researchers, [58], it is not proved here to have a role in distinguishing between the two fracture types. We arrived at the same conclusion for two additional measured parameters, namely Femoral Shaft Diameter (FSD) and Femoral Canal Diameter (FCD), both of which were measured 5 cm distally to the inferior border of the lesser trochanter (Figure 3). It is noteworthy that, despite our study not demonstrating statistically significant differences between genders, the existing literature suggests that the FSD tends to increase proportionately with age. This is attributed to periosteal osteogenesis, effectively mitigating the decline in bone strength associated with diminishing bone mass. Notably, this phenomenon appears to be more pronounced in men [59,60].

The primary objectives of this investigation encompassed the screening of individuals at high risk of experiencing either an initial or contralateral proximal femoral fracture. Subsequently, the study aimed to enhance the preventative measures for this high-risk population and, lastly, to improve the existing surgical techniques. Considering the geometrical aspects of the proximal femur, there exists an opportunity to optimize the design of hip protectors. This optimization seeks not only to meliorate the biomechanical efficacy, but also improve patient acceptance [61,62]. In addition, a rapidly evolving scientific domain focuses on augmenting the proximal femur region in order to prevent subsequent fractures [63]. Identifying geometric factors deemed etiological for femoral neck or trochanteric fractures and integrating them with precise bone mineral density (BMD) measurements of the same area holds the potential for adjustment. Such adjustments could be carried out through techniques like cement and hydroxyapatite augmentation [64] in the pathological area or through the deployment of a prophylactic system [65]. Despite these methods having been exclusively applied to cadaveric specimens thus far, their promise is considerable. Finally, understanding the geometric parameters contributing to fracture causation could assist surgeons not only in increasing the stability of the fixation, but also to restoring the normal biomechanical environment of the hip. This improvement could be achieved by employing prostheses that are appropriate to the geometrical and morphological characteristics of each patient. This includes considerations such as choosing between cemented or uncemented options, selecting a high or low offset, determining the diameter of the body and neck of the implant, and optimizing the Neck Shaft angle [13,66,67].

There exist certain limitations within our study. Despite the fact that a standardized protocol was consistently applied for pelvic radiographs, these were carried out using various radiological machines and by different technicians. Even minor deviations in the imaging process may have had an impact on our recordable parameters. In addition, our study did not incorporate an assessment of patients’ previous medical history and potential risk factors associated with osteoporosis. These particular variables could exert an influence on the measured elements within our study, notably parameters like FSD and FCD. Furthermore, we utilized conventional X-ray imaging instead of quantitative CT scans (qCt). This approach, while offering less precision in parameter assessment, would enhance simplicity and replicability in our screening tool. Our rationale for this choice was to develop a straightforward and easily reproducible screening tool with broad applicability. Ultimately, it is important to note that the size of our sample, comprising patients of Greek origin, was relatively small, predominantly due to the stringent nature of our inclusion and exclusion criteria. Future studies should address these limitations and emphasize Bone Mineral Density (BMD) measurements, as these could possibly predict the fracture type and associated risks.

## 5. Conclusions

The primarily goal of this study was to identify the anatomical parameters within the proximal femur and pelvis that are easily measurable and have the potential to differentiate between fractures occurring in the femoral neck and trochanteric region. Such identified parameters could function as a useful screening tool, enabling the anticipation of the likelihood of future fractures and enhancing the overall outcomes of fracture fixation procedures. Regrettably, our research fell short of achieving this objective, underlining the imperative for further investigations to address this gap in knowledge and contribute to the refinement of clinical practices in fracture management. In this context, researchers could utilize the precise mapping of bone mineral density across the proximal femur region by employing the Dual-Energy X-ray Absorptiometry (DEXA) method and quantitative Computed Tomography (qCT). The accurate characterization, definition, and integration of vital factors encompassing geometrical configuration, bone mineral density, and intrinsic microarchitecture of the hip hold the potential to facilitate precise and early preventive measures against proximal femoral fractures.

## Figures and Tables

**Table 1:** Descriptive statistics, total sample.

		Ν	%
Gender	Women	51	72.9%
	Men	19	27.1%
Tönnis classification	0	1	1.4%
	1	6	8.6%
	2	42	60%
	3	19	27%
	4	3.8	2.9%
		**Mean**	**Standard Deviation**
Age (ys)		80.5	9
Femoral Head Diameter (FHD)		5.4	0.4
Femoral Neck Diameter (FND)		3.8	0.4
Femoral Shaft Diameter (FSD)		3.1	0.3
Femoral Canal Diameter (FCD)		1.6	0.3
Offset Length (OL)		3.8	0.7
Neck Length (NL)		5.2	0.8
Neck Shaft Angle (NSA)		132.1	5.1
Wiberg Angle (WA)		44.9	8.1
Acetabular angle (AA)		34.6	3.8
Hip Axis Length (HAL)		11	0.9

**Table 2:** Analysis by fracture type.

		Femoral Fracture Type				
		Trochanteric (FTF)		Neck (FNF)		
		Ν	%	Ν	%	*p*
Gender	Women	24	80.0%	27	67.5%	0.244
	Men	6	20.0%	13	32.5%	
Tönnis classification	0	0	0.0%	1	2.5%	0.271
	1	4	13.3%	2	5.0%	
	2	16	53.3%	26	65.0%	
	3	8	26.7%	11	27.5%	
	4	2	6.7%	0	0.0%	
PAUWELS	1			3	7.5%	
	2			27	67.5%	
	3			10	25.0%	
AO	31A1,1	4	13.8%			
	31A1,3	2	6.9%			
	31A2,1	1	3.4%			
	31A2,2	10	34.5%			
	31A2,3	9	31.0%			
	31A3,2	2	6.9%			
	31B2,2	1	3.4%			
		**Mean**	**Standard Deviation**	**Mean**	**Standard Deviation**	***p***
Age (ys)		83.0	8.1	78.7	9.3	0.050
Femoral Head Diameter (FHD)		5.3	0.4	5.4	0.3	0.398
Femoral Neck Diameter (FND)		3.7	0.4	3.8	0.3	0.214
Femoral Shaft Diameter (FSD)		3.1	0.3	3.0	0.3	0.387
Femoral Canal Diameter (FCD)		1.7	0.3	1.6	0.3	0.094
Offset Length (OL)		4.0	0.7	3.7	0.7	0.125
Neck Length (NL)		5.3	0.8	5.1	0.8	0.190
Neck Shaft Angle (NSA)		130.9	4.3	133.0	5.6	0.097
Wiberg Angle (WA)		45.8	6.7	44.2	9.0	0.405
Acetabular angle (AA)		34.7	3.4	34.5	4.1	0.858
Hip Axis Length (HAL)		11.1	0.9	11.0	0.9	0.620

## Data Availability

Data are contained within the article.
